# Psychological Safety in Ghana: Empirical Analyses of Antecedents and Consequences

**DOI:** 10.3390/ijerph17010214

**Published:** 2019-12-27

**Authors:** Mavis Agyemang Opoku, Suk Bong Choi, Seung-Wan Kang

**Affiliations:** 1 College of Business, Gachon University, Seongnam 13120, Korea; opokuagyemangm@yahoo.com; 2 College of Global Business, Korea University, 2511 Sejong-ro, Sejong City 30019, Korea

**Keywords:** LMX, education, organizational tenure, conservation of resources theory, psychological safety, voice behavior

## Abstract

This study examines psychological safety as a mediator in the relationship between Leader–Member Exchange (LMX) and voice behavior. Based on the conservation of resources theory, a moderated mediation framework was used to examine human capital investments, specifically employee education and tenure, as boundary conditions of this relationship. The research hypotheses were tested with a sample of 207 employee-supervisor dyads working in a time-lagged design. The study found that psychological safety is an intermediary mechanism through which LMX affects voice behavior. Employees’ level of education negatively moderates the relationship between LMX and psychological safety. Furthermore, the results suggest that organizational tenure accentuates the relationship between LMX and psychological safety, and strengthens the indirect effect of LMX on voice behavior. The theoretical contributions and managerial implications are discussed in addition to directions for future research.

## 1. Introduction

Organizations increasingly require their employees to work collaboratively across disciplines and boundaries to achieve organizational goals [1]. Doing so requires employees to exhibit learning behavior by speaking up, cooperating with others and experimenting with novel ideas [2,3]. While learning has multiple benefits for the organization, the primary beneficiary is the individual because learning constitutes elements of personal growth and development [4]. As learning requires employees to engage in activities voluntarily, it carries potential risks for the individual, including the risk of being seen as disruptive, ignorant, or even incompetent [5,6]. The risks often discourage employees from applying themselves to the learning process, which, in turn, hinders individual and organizational learning [7]. While the most convenient way out may be to simply avoid situations with uncertain outcomes, this may have detrimental consequences—especially in high-risk industries where admitting to errors can prevent major catastrophes [8]. 

Research has found that psychological safety is essential for organizational learning to take place [3]. Thus, there has been a growing body of research seeking to understand the factors that contribute to psychological safety, as well as its outcomes [1]. The origin of the psychological safety construct can be traced back to the seminal work on organizational change by two MIT professors, Edgar Schein and Warren Bennis [9]. In their work, the professors argued that when confronted with changing organizational challenges, psychological safety equips people to feel safe and helps them change their behavior accordingly. Almost three decades later, Schein [10] extended the literature and argued that psychological safety helps overcome the anxiety of learning and helps employees shift their focus away from a mindset of self-preservation and lean more toward the achievement of communal goals. Subsequent works by Kahn [11] and Edmondson [2] helped renew interest in this area. 

In their recent review work, Newman et al. [6] pointed out that most of the empirical work on psychological safety has treated the construct as a mediating mechanism that explains how positive leadership behavior, organizational practices, relational networks, and individual and/or team differences can facilitate positive organizational outcomes. This study examines how the dyadic relationship between the leader and the follower (also known as Leader–Member Exchange (LMX)) affects voice (a learning behavior). By voice behavior, we refer to discretionary workplace behavior that describes the expression of opinions, ideas, concerns and suggestions on work-related issues with the intent to improve the work unit and organization [12]. We investigate psychological safety as the mechanism through which LMX affects voice behavior. 

Although work on the construct of psychological safety has been growing over the past two decades and continues to provide much insight, there are several knowledge gaps in the understanding. A large proportion of the research on psychological safety relies on the social exchange theory and social identity theory [13,14,15] to explain how supportive leadership promotes psychological safety and subsequently influences positive work outcomes. The overconcentration on these two theories limits our theoretical understanding of how psychological safety unfolds. Following the recommendations of Newman et al. [6], we draw on the conservation of resources (COR) theory [16,17] to examine how psychological safety unfolds.

A review of prior empirical studies on psychological safety points to a high concentration in cultures with low power distance and uncertainty avoidance, like the United States. Power distance and uncertainty avoidance are cultural elements that form part of the six dimensions of national culture developed by Hofstede et al. [18]. The power distance dimension describes the extent to which the less powerful persons in a society accept the unequal power distribution [19]. Societies with high power distance tend to accept hierarchical systems without requiring justification. Alternatively, people in low power distance cultures tend to be critical of unequal power distribution. The uncertainty avoidance dimension describes the extent to which people in a society are uncomfortable in situations with uncertain or ambiguous outcomes [19]. To avoid such ambiguity, strong uncertainty avoidance societies adhere to rigid rules, laws, and behavioral codes. Cultures with weak uncertainty avoidance view uncertainty as inherent in life, and people are more receptive to novel ideas [18].

Low power distance cultures are more receptive to learning behavior, such as speaking up so that the absence of psychological safety may not inhibit learning behavior. On the contrary, individuals in high power contexts who speak up, for instance, are more likely to face resistance in the form of social costs, including alienation from the group or negative effects on a career. Extending research on psychological safety to high power cultures can contribute to the predictive validity of psychological safety [6]. Thus, we extend the literature by examining psychological safety in Ghana. Most organizations in Ghana adopt a family culture style that is power-oriented [20]. Managers expect workers to perform their work roles as directed. In return, employees have an expectation that managers will take on a father figure role by caring about subordinates and from whom they can seek approval and guidance at work [20]. Organizational structures tend to be centralized with a hierarchical chain of command [21]. Workers prefer their managers to take the big organizational decisions so far as their interests are not sacrificed [20]. Thus, the Ghanaian organizational style aligns with Hofstede’s [19] high uncertainty avoidance and high power distance cultural dimensions [20]. 

Psychological safety is often presented in the literature as a predictor of learning without much recourse to its boundary conditions. There have been calls for research to pay attention to factors that strengthen or neutralize psychological safety [22]. A similar argument has been made in favor of voice research where Morrison [23] contended that the role of individual-level factors in influencing employee voice behavior had been limited, and the empirical results have been mixed. This is relevant because individual differences influence behavioral outcomes in the workplace. As a response to these issues, this study examines how the interactive effect of LMX and individual attributes can affect psychological safety and voice behavior. Specifically, we focus on the most likely forms of human capital, namely education and organizational tenure, which employees accumulate over the course of their careers [24]. 

Several recent studies suggest that both factors are important in predicting several individuals and workplace outcomes, including career success, job satisfaction, and job performance [25,26], and that they can possibly influence psychological safety and voice behavior. According to the COR theory [16], individuals have limited resources and are highly motivated to protect them. The theory argues that to gain better insight into stressful situations, it is best to focus on the resources that are available to deal with the stressful situation rather than the situation itself [17]. As the role of mediators is often contingent on specific situations and contexts [27], this study uses a moderated mediation framework to investigate the assertion that the mediating effect of psychological safety between LMX and voice behavior will be contingent on an employee’s education level and organizational tenure. 

**Figure 1:**
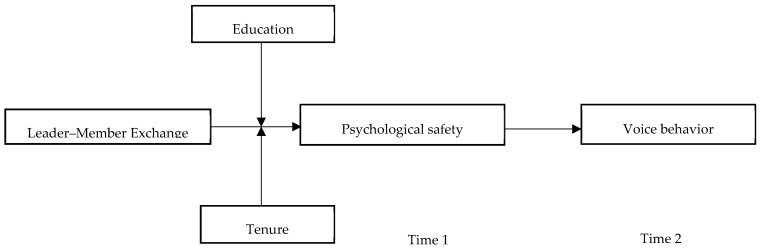
Hypothesized research model.

In sum, this study seeks to address three research objectives. First, we use the COR theory to examine LMX as an antecedent of voice behavior. Second, we examine psychological safety as an important resource that explains why employees working under high LMX are more likely to exhibit voice behavior in a new cultural context. Finally, we use a moderated mediated framework to explore how employee education level and organizational tenure as individual attributes can strengthen or neutralize how LMX facilitates psychological safety, and ultimately, voice behavior. The proposed research model is depicted in Figure 1. By addressing these issues, this study seeks to not only bring clarity to psychological safety literature, but also to provide insights on how leaders can direct managerial policies to improve voice behavior in the workplace. 

## 2. Theoretical Background and Hypotheses 

### 2.1. LMX and Voice Behavior

The quality of the relationship with one’s immediate supervisor is an essential cue that factors in the employees’ decision to speak up. Supervisors are among the main targets of voice behavior because of the power they have over work assignments, salaries and performance ratings. Prior empirical research literature has linked positive leadership to voice behavior [28,29,30]. Originally known as the vertical dyad linkage, LMX was conceptualized in the 1970s, and describes the dyadic relationship between the leader and the follower. LMX is rooted in the principle that leaders create differentiated relationships through the differentiated types of exchange that they have with their followers [31]. High-LMX relationships are characterized by respect, trust, and a sense of mutual obligation that result in an affective attachment for one another [32,33]. In such relationships, both the leader and the follower view the relationship as socio-emotional, going beyond a merely transactional economic exchange [34]. This leads to a reciprocal loop, because when leaders show care and concern for subordinates, it creates stronger leader-follower relationships [35]. Empirically, LMX has been linked to multiple organizational outcomes [34,36,37]. Following the COR theory, we explore the relationship between LMX and voice behavior by examining LMX as a social or relational resource [38] that helps deal with the potential or actual consequences of speaking up. 

The COR theory is a motivational theory that explains how individuals and groups’ strive to obtain, retain, protect, and invest in resources to sustain their resource pool [16,17]. Resources are primarily defined as objects (e.g., work tools, house), personal characteristics (e.g., optimism, self-efficacy), conditions (e.g., seniority, tenure), and energies (e.g., knowledge, time) valued by individuals, or those that serve as a means of attaining these units. Supportive work relationships are condition resources [39] because they facilitate the preservation, sustenance, or depletion of a person’s resources [16]. This suggests that LMX, which has been referred to as the fundamental unit of interpersonal interactions and relations [40], can be a vital resource that employees can invest in to protect valued workplace resources. 

The basic premise of the resource investment principle under the COR theory is that individuals invest in resources to get more resources, to protect against the possible loss of resources, and to recover from losses when they occur [39]. This possibility of loss is implicit in the use of voice. Even when a person offers constructive ideas, there is still an element of risk as speaking up itself suggests a challenge to the existing structures and authorities [12]. In other words, employees who invest in LMX relations may be more adept at dealing with the perceived challenges of speaking up. 

Employees in high-LMX relationships are better positioned to have more access to supervisors and to receive job-relevant knowledge from them [41]. This increased access is likely to translate into a higher frequency of communication and consequently more opportunities to speak up. The benefits of high-LMX for employees include receiving relatively more support and better supervisor responsiveness [33], which signals to employees that they are valued [42]. Such employees enjoy relatively more support and better supervisor responsiveness [43]. Wang et al. [37] suggested that high LMX engenders role expansion. As such, the intrinsic motivation to contribute to the work environment increases positively [44]. By extension, LMX is likely to be associated with the increased use of voice as a means of engaging in proactive behavior to bring about positive organizational change.

Hsiung [44] proposed that high LMX evolves to the level of partnership. Over time, subordinates in such relationships have a better understanding of their supervisor and are able to speak up knowing that their intentions will not be misunderstood. Thus, LMX quality becomes a key factor in the enthusiasm and conscientiousness of the subordinate when it comes to initiating change in the organization. The findings from the LMX literature has linked high-LMX relationships to employee voice behavior [44,45,46]. Based on theory and empirical evidence, we posit the following hypothesis:

**Hypothesis** **1.** *LMX is positively related to voice behavior.*

### 2.2. The Mediating Role of Psychological Safety

Employees take risks every day as they interact with others and navigate change or uncertainty in the workplace [5]. To assess any potential interpersonal risk, a question that employees often ask is, “If I do this here, will I be hurt, embarrassed or criticized?” A negative response to this tacit question informs the employees’ decision to proceed with an intended course of action because it constitutes either the absence of or low psychological safety [3]. Psychological safety is a cognitive state that refers to the feeling of being able to employ oneself without the fear of negative costs to self-image, status, or career [11]. It reflects the perceptions of how others will respond when individuals decide to engage in learning behavior by seeking feedback, questioning issues, reporting mistakes, or experimenting with new ideas [2]. 

Perceptions are important because they inform behavior [47] and Edmondson and Lei [1] contended that employees who feel psychologically safe are willing to contribute ideas and perform effectively at work. Employees who feel psychologically safe may be direct, open, and authentic in specific roles and settings [3]. Psychological safety perceptions are specific to the work environment and not the work role [48]. However, it does not imply an environment free of problems, but rather one where individuals can work toward accomplishing communal goals without having to worry about possible fallouts as they go about the process [3]. 

Because psychological safety relates to the work environment, the leadership style exhibited can set the tone that characterizes the environment. To achieve this, leaders can be intentional, while building relationships and facilitating communication to create work environments in which employees know that engaging in the process of change through self-expression comes at no cost to self-image or status within the work unit. Edmondson [2] identified positive leadership as an antecedent to psychological safety, and the current literature provides empirical support [13,49,50].

The COR theory asserts that the more resources an individual has, the more capable they are of gaining and preserving other resources [39]. Thus, individuals who enjoy the relational and social support of the leader may be more likely to gain valuable work resources [51]. LMX, as the primary unit of interaction, can be a vehicle for both the creation and enhancement of employee psychological safety perceptions. Through LMX, leaders are able to wield positive psychological influence over followers [52]. High LMX creates trusting and supportive relationships that are vital to the perceptions of psychological safety. That is, by providing employees with the flexibility to work, take new initiatives, and even fail at them without having to worry about negative repercussions or retaliation [11]. When employees in high-LMX relationships are given more responsibility, it engenders beliefs that the supervisor trusts their judgments and their ability to handle extra work. This can, in turn, create perceptions that their opinions will be valued, and ultimately, factor in employees’ decision to offer suggestions. 

Subordinates in high-LMX relationships have access to more information. Thus, it seems likely that armed with relevant information, such employees can feel more confident to question or give ideas about work processes. The mutual affect and loyalty characteristic of high-LMX relationships also suggest that when employees offer ideas that are not accepted or implemented, there may be an expectation that criticisms will be constructive and not destructive. Essentially, employees will believe that they will be given the benefit of the doubt and not penalized for communicating upward. 

According to the COR theory, even when individuals are not dealing with any current stressors, they seek to maintain a reservoir of resources to offset potential resource loss in the future [16]. Since the decision to take a risk is one that individuals are routinely confronted with at the workplace, psychological safety is a resource that an employee may attempt to accumulate by investing in high-quality LMX. Uhl-Bien and Maslyn [53] argued that psychological safety is an outcome-related consideration that drives voice behavior. As speaking up is neither required by formal contractual agreements nor recognized by formal reward systems, a conscious effort is made to weigh the consequences before speaking up. As Morrison and Rothman [54] explained, voice behavior involves an implicit suggestion that the status quo is not working optimally and that there is a concern that targets of voice may see voice behavior as a challenge to their authority. Before speaking up, employees make a judgment call that it is safe to do so—that is, they assess whether the potential benefits will outweigh the potential consequences.

One mechanism that is often adopted, while facing a resource loss is the shift in the focus of attention [16]. Thus, by focusing on the possible gains as opposed to losses, individuals can reinterpret a threat as a challenge. Applying this principle to the context of voice, employees may overlook the possible risk of speaking up and instead focus on how speaking up can contribute to LMX and create a reinforcing cycle. While this may be easier said than done, we contend that employees who gain psychological safety through LMX can arguably be better equipped to refocus their attention. Given that LMX is influential in facilitating psychological safety and perceptions of psychological safety affect voice behavior [55,56,57], we expect psychological safety to play a mediating role between LMX and employee voice behavior. Hence, we hypothesize that:

**Hypothesis** **2.** *Psychological safety mediates the relationship between LMX and voice behavior.*

### 2.3. The Interactive Effect between LMX and Employee Education

Education level describes an individual’s academic degrees or credentials [58]. When organizations institute minimum educational requirements, there is an assumption that they are indirectly employing individuals for their cognitive abilities [59]. Ng and Feldman [58] contended that the knowledge provided at school prepares prospective employees to be more competent in their respective vocations and to, eventually, climb up the occupational and organizational ladder faster. Drawing on the COR theory, we argue that education is a valued work resource that may play a vital role in the relationship between LMX and psychological safety. Specifically, we propose that an employee’s education may partially substitute the role of LMX in fostering perceptions of psychological safety, such that the relationship will be stronger when an employee has a low level of education.

Education is often used by firms as an indicator of a potential employee’s productivity or skill level [60]. Thus, the higher an individual’s education level, the more likely they are to occupy a relatively higher position or be promoted quicker [58]. By virtue of the higher positions they occupy in the hierarchy, employees with high education levels may hold a higher status within the work group if other conditions are the same. This higher status often comes with benefits at work, including the privilege to occupy the best office spaces and more power over actions within the work group. Previous research has shown that status is an antecedent to psychological safety [61].

As a selection criterion, firms are willing to pay higher salaries for employees with high education levels because of the implicit assumption that high levels of education are associated with work values like responsibility, honesty, and social relationships that affect citizenship performance, and ultimately, job success [58]. For individuals with a low level of education, this pattern may result in self-doubt with respect to their abilities and capabilities as they are passed over for promotions or observe others with high education levels being given higher entry level positions.

The existing literature on individuals with a low level of education indicates that they do not fare very well in the labor market, and their employment is often characterized by volatility and insecurity [62]. They have a higher risk of losing their jobs and have a harder time re-entering the job market [63]. With advancements in technology, there is a rising demand for high skilled labor, especially as more manufacturing firms opt for automation [64]. For employees with low education levels, this creates an issue of skill obsolescence which often becomes a source of possible social and economic marginalization [63]. Thus, for employees with a low level of education, there can be an inherent lack of psychological safety in the workplace that is not experienced by their counterparts with high education levels. 

The preceding arguments suggest that, because education is a vital work resource, employees with high education levels may not rely on a relational resource, such as LMX, in order to gain psychological security. Alternatively, for employees with low education levels, the relationship with the supervisor serves as a referent standard for their psychological safety. Thus, we propose the following hypothesis:

**Hypothesis** **3.** *Education moderates the relationship between LMX and psychological safety such that the relationship is stronger for employees with lower education level.*

By extension, we argue that an employee’s education level can neutralize the indirect relationship between LMX and voice behavior via psychological safety. The COR theory suggests that individuals use resources to respond to stressful situations and challenges [16]. The gain paradox principle of the COR theory posits that in a situation in which the potential for resource loss is high, the resource gains become even more valuable. This implies that in a high stress situation like the one related to speaking up, having a high level of education becomes a very valuable resource. In contrast, individuals who lack resources are more susceptible to resource loss and increasingly less capable of regaining lost resources [17]. We extend this principle and suggest that relative to workers with a high level of education, those with a low education level may have an exaggerated perception not only of the costs associated with speaking up, but also of their ability to recover from any potential loss.

In an exploratory study on voice, respondents indicated that the fear of retaliation, specifically the fear of losing their jobs or not getting promoted were partly why they did not speak up in the workplace [65]. Given the job insecurity and volatility in the job market for employees with a low level of education [62], we argue that these workers are more likely to harbor such fears. Thus, workers with a low level of education may be unwilling to put themselves in situations or partake in behaviors that can aggravate their perceived or actual job insecurity. 

Ng and Feldman [58] suggested that in addition to skills and knowledge, education equips individuals with work values that engender citizenship behavior. In their introduction of voice to the extra-role behavior literature, Van Dyne and LePine [66] presented voice as a type of citizenship behavior. The work values acquired through education have also been shown to be negatively related to counterproductive work behavior [58], such as withholding vital concerns and information on workplace issues from persons at the workplace with the power to address these issues. As a form of proactive behavior, we believe that employees with high education levels would be more inclined to use voice as a tool to positively contribute to the work unit. 

For employees with high education levels, the higher social status they enjoy within their work unit suggests that their input is likely to be given more recognition and consideration, thus, making them willing to share their knowledge or information with others. For employees with a low level of education, on the other hand, speaking up may not be met with the same level of recognition. Thus, the quality of the relationship with the leader would be especially important and may factor in their decision to express their opinions. This suggests that the indirect effect of LMX on voice via psychological safety will be stronger when the employee’s education level is low rather than high. Therefore, we propose that:

**Hypothesis** **4.** *Education moderates the indirect effect of LMX on voice behavior via psychological safety such that the indirect relationship is stronger for employees with lower education level.*

### 2.4. The Interactive Effect between LMX and Organizational Tenure

Organizational tenure refers to the period of time that an employee spends at an organization [67]. Over time, tenure results in the acquisition of practical knowledge as employees accumulate more experience, become better acquainted with work processes, and become more adept at performing diverse roles in the organization [68]. Unlike education, tenure as a human capital investment is more precise, because it generates higher returns the longer an employee remains with an organization [26]. The extant empirical literature points to variability in the role of tenure in facilitating multiple cognitive states, including affective commitment and job satisfaction [69,70]. Specific to psychological safety, organizational tenure has been linked to an individual (r = 0.27) and team psychological safety (r = 0.26) [2,71], thus, lending credence to the possibility of organizational tenure as a moderator in the LMX and psychological relationship safety. 

Employees with longer tenure may be more likely to feel psychologically safe. We contend that as employees spend more time at an organization and gain experience, they become more knowledgeable about the firm in general [72]. Therefore, employees with longer tenure, are more likely to understand the boundaries within which they are allowed to operate, as well as the consequences that would emerge if they deviate from these implicit or explicit rules. In comparison, newer employees go through an adjustment phase of socialization where they have to grapple with role clarity, self-efficacy, acceptance by organizational insiders and an understanding of the organizational culture [73]. Psychological safety is likely to develop over time as clearer and predictable organizational patterns are observed and understood. In the absence of clearly defined boundaries of norms, people feel unsafe and withdraw as a self-protective mechanism [11]. 

In his conceptualization of psychological safety, Kahn [11] presented four antecedents (i.e., interpersonal relationships, group dynamics, organizational norms, and management styles and processes) that directly impact individual perceptions of psychological safety. These factors have an implicit time element as individuals need time to build trust in their relations, become cognizant of the unconscious roles taken up by group actors or become familiar with organizational procedures, that is organizational norms, management styles, and processes. Specific to the organizational factors, Kahn [11] argued that knowledge of organizational norms predicts one’s ability to operate within the norms and not stray from the shared expectations of acceptable behavior. Management styles and processes are explained as constituting an environment that reinforces employees’ behavior by affording employees the opportunity to experiment with new work techniques. Both require first-hand knowledge and by implication, tenure with the organization in order to fully appreciate norms and the managerial environment to form perceptions of psychological safety. Based on the preceding arguments, we predict that:

**Hypothesis** **5.** *Organizational tenure moderates the relationship between LMX and psychological safety such that the relationship is stronger for employees with longer organizational tenure.*

Exhibiting voice behavior requires a latent voice opportunity [74]. This means that an individual must have an idea, perspective, or concern that is worth sharing or communicating [12]. Once this opportunity exists, the decision to voice that opinion occurs if there is a fundamental motivation to do so [12]. Morrison [23] noted that this includes an employee having a sense of obligation to positively contribute to the organization. Employees who have spent more time at an organization make greater investments made in the organization, and these investments result in them being more committed to the success of the organization [67]. We posit that the more time that employees spend at an organization, the greater their tendency to offer constructive input and speak up. 

The resource caravan principle of the COR theory proposes that resources do not exist in isolation, but instead, travel in groups for both individuals and the organization [39]. As resources can be created through nurturance and learned behavior, the resource caravan may explain the often high correlation found among valued work resources, such as self-esteem and self-efficacy [17]. Applying this principle to the psychological safety context, we contend that with increased tenure, a resource caravan effect can be activated such that the influence of tenure would move beyond a moderating effect on the relationship between LMX and psychological safety to a conditional indirect effect on voice behavior.

Employees worry that, however, constructive their suggestions or ideas may be, they could be tagged as being problematic and attract negative career consequences [74]. Especially at the initial stage of tenure, employees believe that they are at a learning stage, and thus, lack the credibility to voice their opinions [65]. New employees tend to believe that they are yet to prove their worth, and thus, managers are not very receptive to their ideas [75]. This results in an unwillingness to express their opinions because of the belief that one’s ideas will not be taken seriously [12]. Thus, employees with longer tenure are often more willing to take the risk and speak up [23]. The extant empirical data support this assertion by linking organizational tenure to voice behavior [7]. 

Voice behavior is constructive and purposeful in its intent, wherein an individual speaks up only when he or she believes that doing so will bring about an improvement. As organizational tenure increases, employees get better acquainted with formal power structures [76], and this allows them to be more intentional with when, where, and whom to direct their voice to effect the necessary changes. As employees gain experience over time at an organization, they gain a better understanding of work processes that help them avoid making careless mistakes [26]. Thus, the voice of an employee with a longer tenure is more likely to receive attention than that of a newcomer. Finally, organizations see organizational tenure as a proxy for loyalty and reward employees with long tenure with opportunities for career growth, including training and development [26]. Therefore, the need to reciprocate the rewards received may explain why employees with long tenure are more inclined to speak up. In sum, we argue that the organizational tenure will factor into an employee’s decision to engage in voice behavior. Thus, we propose the following hypothesis:

**Hypothesis** **6.** *Tenure moderates the indirect effect of LMX on voice behavior via psychological safety such that the indirect relationship is stronger for employees with longer organizational tenure.*

## 3. Method

### 3.1. Sample and Procedure

Participants in this study comprised of workers from manufacturing firms located in a major city in Ghana. The data were collected by administering a paper-and-pencil questionnaire to all the participants. Approval was obtained from management before data collection. Participation by both supervisors and employees were voluntary, and participants were assured of the confidentiality of their responses. Following previous literature on LMX and voice [77], the questionnaire was administered to 250 employees and 26 supervisors in two waves with a two-week interval over four weeks. This was done to use a temporal and proximal approach to mitigate the possibility of common method bias [78]. 

Employees completed a survey measuring their LMX, education, organizational tenure, and psychological safety. Supervisors evaluated employee voice behavior with the second survey in wave two. Since LMX takes time to develop and have an impact, we only included the responses of employees who had a dyadic tenure with a supervisor for a minimum of six months. A final sample size of 207 matched dyads remained after we deleted unmatched employee-supervisor pairs and responses with missing data. This corresponded to a response rate of 82.8%. The sample of 207 employees included 106 females (51.2%) and 101 males (48.8%). The average organizational tenure was 3.26 years (SD = 2.24). All participants were full-time employees and the average period of education was 14.46 years (SD = 2.08). 

### 3.2. Measures

#### 3.2.1. Leader–Member Exchange

We used the seven-item scale by Graen and Uhl-Bien [32] to measure LMX. The response scale was a five-point Likert scale (1 = strongly disagree, 5 = strongly agree). A sample item was, “I know where I stand with my leader and I usually know how satisfied my leader is with what I do”. The Appendix A Cronbach’s alpha was 0.87.

#### 3.2.2. Psychological Safety

We used the three-item scale by to May, Gilson, and Harter [79] measure psychological safety. The response scale was a five-point Likert scale (1 = strongly disagree, 5 = strongly agree). A sample item was, “The environment at work is threatening (r).” The Appendix A Cronbach’s alpha was 0.71.

#### 3.2.3. Voice Behavior 

We used the three-item scale by Burris [80] to measure voice behavior. The response scale was a five-point Likert scale (1 = strongly disagree, 5 = strongly agree). A sample item was, “This employee communicates his/her opinions about how to make this team better even if others disagree.” The Appendix A Cronbach’s alpha was 0.81.

#### 3.2.4. Tenure

We measured tenure as a continuous variable representing the amount of time (in years) an employee has worked at an organization [67]. 

#### 3.2.5. Education 

Education was measured as the number of years an employee had received formal education [81].

#### 3.2.6. Control variables

We controlled for employee age and gender in the analysis because these variables have previously been linked with voice behavior and have been statistically controlled for in multiple empirical psychological safety and voice studies [7,61]. Age was measured as the chronological age of employees in years. Gender was dummy coded as 1 for female and 0 for male. 

### 3.3. Analyses

We used STATA 14.1 to conduct hierarchical regression analysis [82] to test our research hypotheses. Prior to conducting the analysis, all the variables that defined the product terms were mean-centered. This was to address multicollinearity issues that were resolved as the variance inflation factor of all variables in the regression analyses fell well below the acceptable standard of 10. We followed the recommendations of Preacher and Hayes [83] in using the bootstrapping approach to test the mediation hypothesis. The moderated mediation hypotheses were analyzed by calculating the index of moderated mediation introduced by Hayes [84]. 

## 4. Results

### 4.1. Descriptive Statistics 

The mean, standard deviation, and zero-order correlations of all variables are summarized in Table 1. The internal reliability of all the measures fell within the acceptable standards (0.71–0.87). As expected, there was a significant correlation between LMX and voice behavior (r = 0.30, *p* < 0.001). LMX and education had a significant correlation with psychological safety (respectively, r = 0.40, *p* < 0.001; r = 0.19, *p* < 0.01).

### 4.2. Confirmatory Factor Analysis and Validity Check 

Before testing the research model, we carried out confirmatory factor analysis (CFA) to test the psychometric validity of the three multi-item measures (i.e., LMX, psychological safety, and voice behavior). The baseline 3 factor model indicated satisfactory fit with the data. The comparative fit index (CFI) was 0.99 with the Tucker-Lewis index (TLI) and root-mean square error of approximation (RMSEA) as 0.99 and 0.03, respectively. Again, the normed chi-square value was 1.18 (χ^2^ = 73.10, df = 62; normed chi-square = 73.10/62) below the standard of 3.00 [85]. As a further test, we set three alternative models and ran the chi-square comparison test between the baseline model and the alternative models. All the alternative models differed significantly from the baseline model and showed relatively weaker model fit indicators as presented in Table 2.

Next, we calculated the average variance extracted (AVE) and composite reliability of the latent variables, which were all above the acceptable standard of 0.50 and 0.70, respectively [86]. Thus, we determined that the focal variables explained variance beyond variance, due to measurement error [87]. In addition, the square root of the AVE for each construct was greater than the construct’s highest correlation involving any other construct in the model. Altogether, all these indicators provide evidence of convergent and discriminant validity. 

### 4.3. Hypotheses Testing

Hypothesis 1 posited a positive relationship between LMX and voice behavior. The regression results in Table 3 support this hypothesis (β = 0.32, *p* < 0.001; Model 6). Hypothesis 2 predicted that psychological safety mediates the relationship between LMX and voice behavior. The results of the bootstrapping method, which does not rely on the normal sampling distribution assumption show a 0.10 coefficient, and the 95% CI with 10,000 times bootstrapped samples did not include zero (0.024, 0.195). Therefore, we determined that Hypothesis 2 is supported.

**Figure 2:**
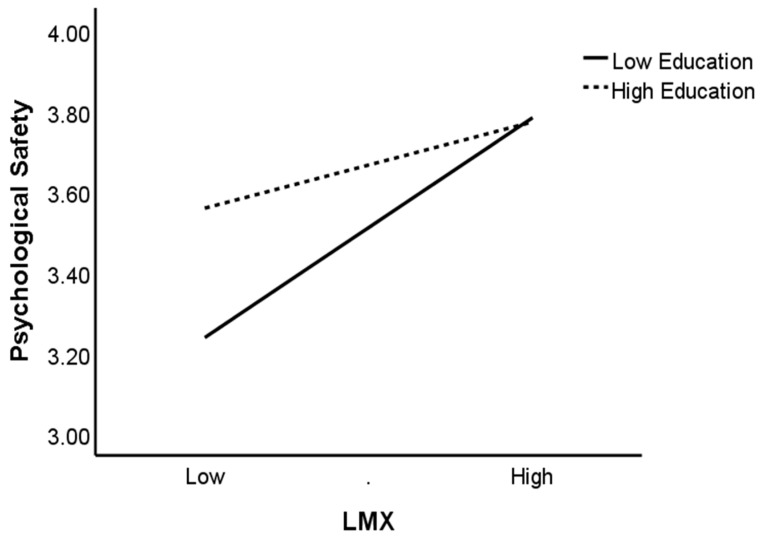
The moderating effect of education level on the relationship between LMX and psychological safety.

Hypothesis 3 postulated that education level would moderate the relationship between LMX and psychological safety negatively. Model 3 of Table 3 showed that the interaction of LMX and education level has a significantly negative relationship with psychological safety (β = −0.14, *p* < 0.05; Model 3). Following Aiken and West [82], we depicted the interaction (Figure 2) by using one standard deviation (SD) above and below the mean education level to represent high and low education levels. As expected, the simple slope test showed that for employees with low education level, the relationship between LMX and psychological safety is stronger (simple slope = 0.46, t = 5.59, *p* < 0.001). In contrast, for employees with higher education levels, the effect of LMX on psychological safety is weaker and insignificant (simple slope = 0.18, t = 1.95, ns). Since these two simple slopes are not the same, these results reconfirm Hypothesis 3. 

To examine the conditional indirect effect of LMX on voice behavior through psychological safety for employees with higher and lower education levels, we used the SPSS macro Process by Hayes [84]. The results of the analyses using 10,000 times repeatedly bootstrapped samples suggest an index of moderated mediation value of −0.02, which is not statistically significant (percentile 95% confidence interval of −0.047 to 0.001). Thus, we determined that Hypothesis 4 is not supported. 

**Figure 3:**
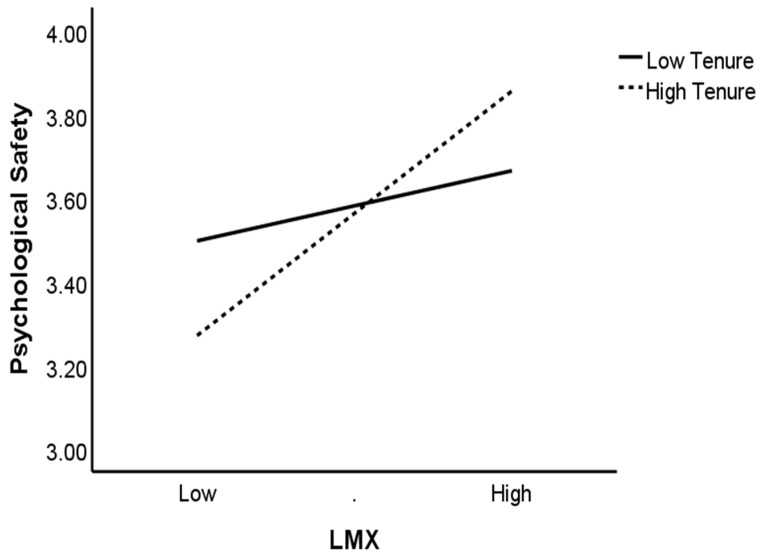
The moderating effect of tenure on the relationship between LMX and psychological safety.

Hypothesis 5 posited that organizational tenure moderates the relationship between LMX and psychological safety positively. This hypothesis is supported by the regression results presented in Model 4 of Table 3 (β = 0.23, *p* < 0.01; Model 4). Next, we conducted the simple slope plotting for tenure at one SD above and below the mean, which is shown in Figure 3, and the simple slope test. As predicted, the relationship between LMX and psychological safety is significantly stronger (simple slope = 0.49, t = 7.41, *p* < 0.001) for employees with high tenure, but weaker and insignificant (simple slope = 0.14, t = 1.79, ns) for low tenure employees. Since these two simple slopes are not the same, these results reconfirm Hypothesis 5.

Hypothesis 6 predicted that the mediated relationship between LMX and voice via psychological safety would be conditional on employee tenure. The results of the analyses using 10,000 times repeatedly bootstrapped samples produced an index of moderated mediation value of 0.02, which is statistically significant (percentile 95% confidence interval of 0.004 to 0.047). We also calculated the indirect effects at high and low level of tenure (i.e., one SD below and above the mean). Under low tenure, the conditional indirect effect (0.04) is lower and insignificant (95% CI [−0.003, 0.105]), but under high tenure, the conditional indirect effect (0.14) is stronger and significant (95% CI [0.029, 0.273]). Since both conditional indirect effects were significantly different, we determined that Hypothesis 6 was supported.

## 5. Discussion

Psychological safety has been presented in previous research as a resource that is valuable and that factors in an employee’s decision to engage in learning behavior. This study focused on voice behavior which describes the extent to which an employee’s act of speaking up and communicating upward with concerns, suggestions, or ideas has vital implications for an organization’s performance and sustained survival [23]. As the fundamental unit of interpersonal interactions and relations in the workplace, LMX is an essential relational resource that enhances employee voice behavior. Within the framework of the COR theory, this study examines the mediating effect of psychological safety on the relationship between LMX and voice behavior. We found that as an employee’s education level increases, the effect of LMX on psychological safety attenuates. Conversely, the relationship is strengthened as organizational tenure increases. The mediated relationship between LMX and voice behavior via psychological safety is conditional on the employee’s human capital investments, specifically organizational tenure. 

### 5.1. Theoretical Contributions

This study contributes to the existing literature in multiple ways. Our initial contribution is to serve as a robustness test of previous literature that has theoretically and empirically linked LMX to voice behavior through psychological safety. The Ghanaian setting of our study provides an opportunity to ascertain the predictive validity of both LMX and psychological safety on voice behavior in a high power distance society. The significantly positive results align with extant literature in other high power distance cultures [88]. These findings provide useful insights because they show that even in high power distance cultures where speaking up is not the norm or expected behavior, LMX can be crucial in encouraging employees to speak up. 

We extended the theoretical understanding of the hypothesized relationships by grounding our research in the COR theory. As a desired work relational resource, LMX has been associated with increased supervisor satisfaction and decreased health complaints of subordinates [51]. Additionally, our study found that by investing in LMX, employees are able to build psychological safety, another invaluable resource that helps protect against any perceived or actual consequences that may accompany voice behavior. As the COR theory [17] explains, individuals seek to retain a reservoir of resources even when they do not currently deal with stressors to protect against possible future resource losses. 

Furthermore, we examined individual-level boundary conditions—an under-researched area—that may interact with LMX to affect psychological safety and voice behavior. The study focuses on the two forms of human capital that employees are likely to accumulate over the course of their careers (i.e., employee education level and organizational tenure). We proposed and found support for our assertion that the role of the high LMX in predicting perceptions of psychological safety was more salient for employees with low education level. We argue that education is often used by firms as an indicator of a potential employee’s productivity or skill level and employees with a high education level tend to occupy higher hierarchical positions. Therefore, these individuals may not be very reliant on the relational resource to feel psychologically safe, especially in our high-power distance context where occupying a high position would be associated with a higher status. 

However, the data did not support our hypothesis that the indirect effect of LMX on voice via psychological safety will also be contingent on the employee’s education level. The unsupported results of hypothesis 4 may occur because of the relatively low education level of the sample. Although the 14.64 average years of formal education corresponds to a Higher National Diploma (HND) which is equivalent to the first two years of a Bachelor’s degree, 47 percent of the participants had an HND qualification or lower. Accordingly, our prediction that the indirect relationship between LMX and voice behavior will be contingent on subordinates’ education level was not supported.

For organizational tenure, a more specific form of human capital investment that generates higher returns the longer an employee stays with an organization, the results showed that the relationship between LMX and psychological safety was strengthened for employees with high organizational tenure. We contend that because newer employees tend to be in an adjustment phase and may not be very familiar with organizational structures and procedures, the interactive effects between LMX and tenure on psychological safety will be more effective for workers with longer tenure as opposed to newer employees. Although preliminary evidence indicates that when incumbent employees speak up, they receive stronger voice recognition from supervisors than newcomers [75], researchers have not examined the role that organizational tenure may play in voice expression. Therefore, we extend the extant literature by presenting tenure as a boundary condition of voice behavior. Our study provides theoretical explanation and empirical evidence uncovering how the identified mediating role of psychological safety between LMX and voice will be contingent on the employee’s organizational tenure. These results support previous research that linked individual differences in behavioral outcomes [89].

### 5.2. Managerial Implications

Overall, our study sought to provide managers with better insights on the factors that influence employee psychological safety and encourages employees to speak up. Our findings point to the importance of good quality relationships with leaders. Thus, managers should invest in building healthy relationships with subordinates through the show of support, loyalty, and respect. This means that the importance of cultivating a good relationship with lower-level employees can be incorporated into training sessions for supervisors. Prior research has shown psychological safety to be related to knowledge sharing, performance, learning behavior, and decision quality at the individual, team and organizational level [1]. Thus, it is in the best interest of organizations and leaders to encourage psychological safety through high LMX. This study also provides a better understanding of how leaders can direct their efforts. The findings indicate that managers need to pay greater attention to their employees with low education level where they are likely to be more effective. In many organizations, employees with low education level tend to hold relatively lower positions and possibly have a lower status within the work unit. However, they also tend to hold frontline positions where their voice would be necessary to gain timely and appropriate feedback on the implementation of new technology or from clients.

Our findings also suggest that organizations should create non-threatening work environments. An extrapolation from the study suggests that newcomers may not feel psychologically safe because it takes time for them to adjust to organizational norms. One way of addressing this would be to streamline and clearly communicate organizational structures and procedures. This can send clear and consistent signals to employees, especially newcomers, on the boundaries within which they are allowed to operate. By institutionalizing routine organizational practices, such as formal evaluations, employees may also be afforded the opportunity to communicate upward with their managers [90]. 

### 5.3. Limitations and Future Research

This study has limitations that should be addressed in future research. Whereas, this study examined the role of education and organizational tenure, the mean number of years of formal education and tenure was relatively low (i.e., 14.64 and 3.26 years, respectively). Thus, future research must analyze data from industries that require employees to have a higher educational level or from organizations with higher employee tenure. By using a sample that consisted solely of workers in the manufacturing sector, generalizability remains limited. Inferences from our analyses should be made with caution as the results may not be applicable to other industries or work contexts. 

Psychological safety was originally theorized as an individual-level construct [11]. However, it was later defined as a shared team belief about how safe it is to take the interpersonal risk [2]. While Frazier et al. [48] argued that both seminal works cited above should be seen as complementary rather than competing sides of the same construct, we encourage further studies to treat psychological safety as a team-level variable. Although the study sought to reduce common method bias with the use of time-lagged data, LMX and psychological safety were all measured by the employee at Time 1, meaning that the responses are still subject to bias. To address this, future research must measure the independent and mediator variables separately and consider longitudinal studies to gain a better understanding of how these constructs evolve over time. 

Although we conducted the study in a high-power distance country, we did not specifically test the effects of cultural elements, such as power distance, collectivism or uncertainty avoidance in our study. Doing so would also contribute to our understanding of the literature. Finally, the supporting empirical results for the moderating role of employee education level and tenure suggests that future research must investigate the role of other individual resources, such as career identity on the proposed relationships. 

## 6. Conclusions

Using the COR theory, our study investigates the effect of LMX on voice behavior. Our findings reinforce psychological safety as a mediating mechanism through which LMX facilitates voice behavior. We examine the role of two forms of individual resources that can impact the relationship. The results point to LMX as important for employees with low education levels in fostering psychological safety. The study also highlights the role of organizational tenure in strengthening the effect of LMX on psychological safety and ultimately, their voice behavior. Despite the limitations, the study provides insights that can help leaders direct their efforts in increasing the psychological safety and the subsequent voice behavior of employees. 

## Figures and Tables

**Table 1:** Means, standard deviations, correlations, and reliabilities.

Variable	M	SD	1	2	3	4	5	6	7
1. Age	32.10	5.96	-						
2. Sex (0 = M; 1 = F) ^a^	0.51	0.50	−0.10	-					
3. LMX	3.88	0.59	0.04	−0.07	0.87				
4. Education ^b^	14.64	2.08	0.40 ***	−0.05	0.17 **	-			
5. Tenure ^c^	3.26	2.24	0.16 **	0.00	0.01	0.03	-		
6. Psychological Safety	3.57	0.52	0.03	−0.00	0.40 ***	0.19 **	0.00	0.71	
7. Voice Behavior	3.73	0.73	0.01	−0.04	0.30 ***	−0.02	0.06	0.29 ***	0.81

Note: n = 207; Internal reliabilities are reported in the diagonal; ^a^ M = male; F = female, ^b^ Education = years of education; ^c^ Tenure = years worked at current organization, ** *p* < 0.01; *** *p* < 0.001 (two-tailed test).

**Table 2:** Model fit statistics for measurement models.

Measurement Model	Χ^2^	df	*p*-Value	CFI	TLI	RMSEA	Δχ^2^	Δdf
Baseline (hypothesized) three factor model	73.10	62	0.158	0.99	0.99	0.03		
Alternative 1 (two factor model) ^1^	191.80	64	0.000	0.87	0.84	0.10	118.70 ***	2
Alternative 2 (two factor model) ^2^	153.84	64	0.000	0.91	0.89	0.08	80.74 ***	2
Alternative 3 (one factor model) ^3^	349.60	65	0.000	0.71	0.65	0.14	276.50 ***	3

Note: n = 207. *** *p* < 0.001 (two-tailed test). ^1^ Two factor model with psychological safety and employee voice behavior on the same factor. ^2^ Two factor model with LMX and psychological safety on the same factor. ^3^ One factor model with LMX, psychological safety and employee voice behavior on the same factor.

**Table 3:** Hierarchical multiple regression for psychological safety and voice behavior.

	Psychological Safety				Voice Behavior		
	M1	M2	M3	M4	M5	M6	M7
**Controls**							
Sex	0.00	0.03	0.04	0.00	−0.03	−0.02	−0.01
Age	0.03	−0.04	−0.05	−0.06	0.01	0.02	0.04
**Independent Variable**							
LMX		0.38 ***	0.36 ***	0.34 ***		0.32 ***	0.24 **
**Moderating Variables**							
Education		0.14	0.15 *	0.11		−0.08	−0.11
Tenure		−0.00	0.03	−0.01		0.06	0.06
**Interaction**							
LMX x Education			−0.14 *				0.01
LMX x Tenure				0.23 **			−0.08
**Mediator**							
Psychological Safety							0.24 **
**Model Fit**							
F	0.07	8.64 ***	8.11 ***	9.72 ***	0.13	4.59 ***	4.29 ***
R^2^	0.00	0.18	0.20	0.23	0.00	0.10	0.15
Adj R^2^	−0.01	0.16	0.17	0.20	−0.01	0.08	0.11
F_inc_		14.34 ***	4.68 *	12.64 ***^a^		7.55 ***	3.51 *
ΔR^2^		0.18	0.02	0.03		0.09	0.05

Note: n = 207. Entries are standardized regression coefficients. ^a^ = F-test compared with Model 2. * *p* < 0.05; ** *p* < 0.01, *** *p* < 0.001 (two-tailed test). Standardized regression coefficients reported.

## Appendix A

**Leader–Member Exchange (α = 0.87)** [32]

1. I know where I stand with my leader, and I usually know how satisfied my leader is with what I do.
2. My leader understands my job problems and needs.
3. My leader recognizes my potential.
4. Regardless of how much formal authority my leader has built into his or her position, there is a good chance that my leader would use his or her power to help me solve problems in my work.
5. Again, regardless of the amount of formal authority my leader has, there is a good chance that he or she would “bail me out” at his or her expense?
6. I have enough confidence in my leader that I would defend and justify his or her decision if he or she were not present to do so.
7. I have an effective working relationship with my leader.

**Psychological Safety (α = 0.71)** [79]

1. I’m not afraid to be myself at work.
2. I am afraid to express my opinions at work (r).
3. The environment at work is threatening (r).

**Voice behavior (α = 0.81)** [80]

1. This employee speaks up and encourages others in this group to get involved in issues that affect the team.
2. This employee communicates his/her opinions about how to make this team better even if others disagree.
3. This employee develops and makes recommendations concerning issues that affect this team.
